# Physical, language, neurodevelopment and phenotype-genotype correlation of Chinese patients with Mowat-Wilson syndrome

**DOI:** 10.3389/fgene.2022.1016677

**Published:** 2022-11-03

**Authors:** Lihua Wu, Jianhong Wang, Lei Wang, Qi Xu, Bo Zhou, Zhen Zhang, Qi Li, Hui Wang, Lu Han, Qian Jiang, Lin Wang

**Affiliations:** ^1^ Department of Medical Genetics, Capital Institute of Pediatrics, Beijing, China; ^2^ Department of Child Health Care, Children’s Hospital, Capital Institute of Pediatrics, Beijing, China; ^3^ Department of General Surgery, Children’s Hospital, Capital Institute of Pediatrics, Beijing, China; ^4^ Institute of Basic Medicine, Chinese Academy of Medical Sciences & School of Basic Medicine, Peking Union Medical College, Beijing, China

**Keywords:** physical development, language assessment, Mowat-Wilson syndrome, phenotype-genotype correlation, *ZEB2*

## Abstract

**Background:** To report detailed knowledge about the clinical manifestations, genetic spectrum as well as physical, language, neurodevelopment features and genotype-phenotype correlations of Chinese patients with Mowat-Wilson syndrome (MWS).

**Methods:** We retrospectively collected and analyzed clinical data for twenty-two patients with molecularly confirmed diagnoses. We used Gesell Developmental Schedules (GDS) to assess their neurodevelopment and the Diagnostic Receptive and Expressive Assessment of Mandarin-Infant & Toddler (DREAM-IT) to evaluate their language ability and compared the data with the two types of underlying pathogenic variations.

**Results:** The height and weight of all patients were below the 75th percentile, and microcephaly was observed in 16 of 22 patients (72.7%). Four patients carrying chromosome deletions encompassing the *ZEB2* gene were more severely affected. All MWS patients exhibited better performance in cognitive play and social communication than in receptive and expressive language. In the receptive language area, the types of words that children with MWS understood most were nouns, followed by adjectives and verbs.

**Conclusion:** This study delineated the phenotypic spectrum of the largest MWS cohort in China and provided comprehensive profiling of their physical, language, neurodevelopment features and genotype-phenotype correlations.

## Introduction

Mowat-Wilson syndrome (MWS, OMIM 235730) is a rare autosomal dominant disorder caused by a heterozygous deletion or loss-of-function variant of the *ZEB2* gene (zinc finger E-box binding homeobox 2). Clinical manifestations of MWS are characterized by typical facial dysmorphism, moderate to severe intellectual disability (ID), global developmental delay, and multiple congenital anomalies, which may involve short stature, microcephaly, Hirschsprung disease (HSCR), corpus callosum agenesis, malformations of the brain, genitourinary anomalies (most commonly hypospadias), and congenital heart defects (Mowat et al., 2003). Since the first description in 1998, more than 350 MWS individuals have been reported (Garavelli et al., 2017; Ivanovski et al., 2018; Ivanovski et al., 2020), and the incidence of the syndrome is estimated to be 1:50,000–70,000 live births (Ghoumid et al., 2013).

The *ZEB2* gene is a member of the *ZEB* family of zinc finger transcription factors, located on chromosome 2q22-q23, originally identified as a transcriptional corepressor for the transforming growth factor *β* signaling pathway through binding to SMAD proteins. To date, more than 220 pathogenic variants of *ZEB2* have been associated with MWS according to the Human Gene Mutation Database (HGMD, http://www.hgmd.cf.ac.uk). Among them, point mutations have been identified in 70%–80% of MWS patients; full or partial deletion of the gene accounts for another 15%–50%, resulting in either truncated or absent protein products, and missense mutations are present in a small percentage (less than 2%) (Ivanovski et al., 2018). Although no obvious genotype-phenotype correlation has been established, MWS patients with large deletions of *ZEB2* are suspected to have more severe phenotypes (Cerruti Mainardi et al., 2004; Ishihara et al., 2004).

In addition to a distinctive facial appearance, most patients with typical MWS have moderate to severe intellectual disabilities, accompanied by severe language impairment (Cordelli et al., 2021). As reported (Ivanovski et al., 2018; Ho et al., 2020), speech in MWS patients rarely extends to more than a few words, and many even have an absence of speech. Meanwhile, receptive language skills are believed to be less affected, and patients with absent speech can sometimes communicate successfully using alternative methods, such as gestures. Systematic language assessments of MWS patients to date have been sparse, and the correlation between its severity and the type of *ZEB2* variation remains unclear. Herein, a comprehensive profile of the physical, language and neurodevelopment features in twenty-two MWS patients of Chinese ancestry was attempted. We used the GDS to assess their neurodevelopment and DREAM-IT questionnaires to evaluate their language ability. To our knowledge, this is the largest case series in China for detailed assessment of phenotypic, genetic spectrum and phenotype-genotype correlations, which will provide important guidance for appropriate intervention and patient care in the future.

## Materials and methods

### Study cohort

Altogether twenty-two MWS patients of Chinese ancestry were recruited, whose molecular confirmation of ZEB2 variations was identified. Basic clinical information was obtained through a web-based highly detailed questionnaire.

The number of involved systems was calculated for each patient according to their neurological, cardiac, gastrointestinal tract, urogenital/renal, eye, teeth, musculoskeletal and behavioral phenotypes. This study was approved by the Ethics Committee of Capital Institute of Pediatrics (SHERLL 2013039). Written informed consent was obtained from the guardians of all of the patients.

### Assessment of neurodevelopment

The Gesell Developmental Schedules (GDS) (Gesell and Amatruda, 1941) is a classic international child development scale that examines five domains: adaptability, gross motor, fine motor, language, and social personality. The Chinese version of the GDS has been modified by the Chinese Pediatric Association with good validation against the Chinese reference population (Song and Zhu, 1987; Yang, 2016). GDS is widely used not only for assessing the neuropsychological development of children aged 0–6 years but also serves as one of the standardized methods for assessing the intellectual impairment of children aged 0–6 years (Chen et al., 2020). GDS is of diagnostic and rehabilitative significance for children who may have neuropsychological developmental delays (Liu et al., 2016). The results are expressed in terms of the developmental quotient (DQ), which is calculated as DQ = developmental age (DA)/chronological age (CA) × 100. Children with a DQ ≥ 85 were deemed normal in terms of developmental status, whereas those with a DQ < 40 were diagnosed as having severe neurodevelopmental disabilities. Based on the degree of neurodevelopmental disability, the DQ scores were divided into mild (55 ≤ DQ < 75), moderate (40 ≤ DQ < 55), severe (25 ≤ DQ < 40), and suspected (75 ≤ DQ < 84) neurodevelopmental disability.

### Assessment of language development

The Diagnostic Receptive and Expressive Assessment of Mandarin-Infant & Toddler (DREAM-IT) is a standardized language test used to evaluate children’s language development levels and has good reliability and validity (De Villiers et al., 2018; Liu, 2019). DREAM-IT has 4 dimensions, including receptive language, expressive language, cognitive play and social interaction. The test is standardized on normal children 0–3 years old. The developmental age refers to the norm of children aged 0–3, and the results are expressed by the developmental quotient (DQ), which is calculated as DQ = developmental age (DA)//chronological age (CA) × 100. A quotient of greater than or equal to 80 points indicates no delay. In addition, DREAM-IT also calculates word number and types and evaluates the consonant development of the tested children.

### Quality control

Three experienced psychologists who have obtained evaluation qualification certificates participated in this study. Before the formal investigation, conformance tests of the GDS and DREAM-IT were conducted, and the intragroup correlation coefficients were both above 0.9. For the same MWS child, the two tests were completed by one evaluator within 24 h, and the original score was recorded.

### Statistical analysis

Statistical analysis was performed with SPSS Version 12 (SPSS China, Beijing, China). Differences in the listed categorical characteristics between patients were assessed using Student’s t test. *p* < 0.05 was considered statistically significant.

## Results

### Cohort analysis

**FIGURE 1:**
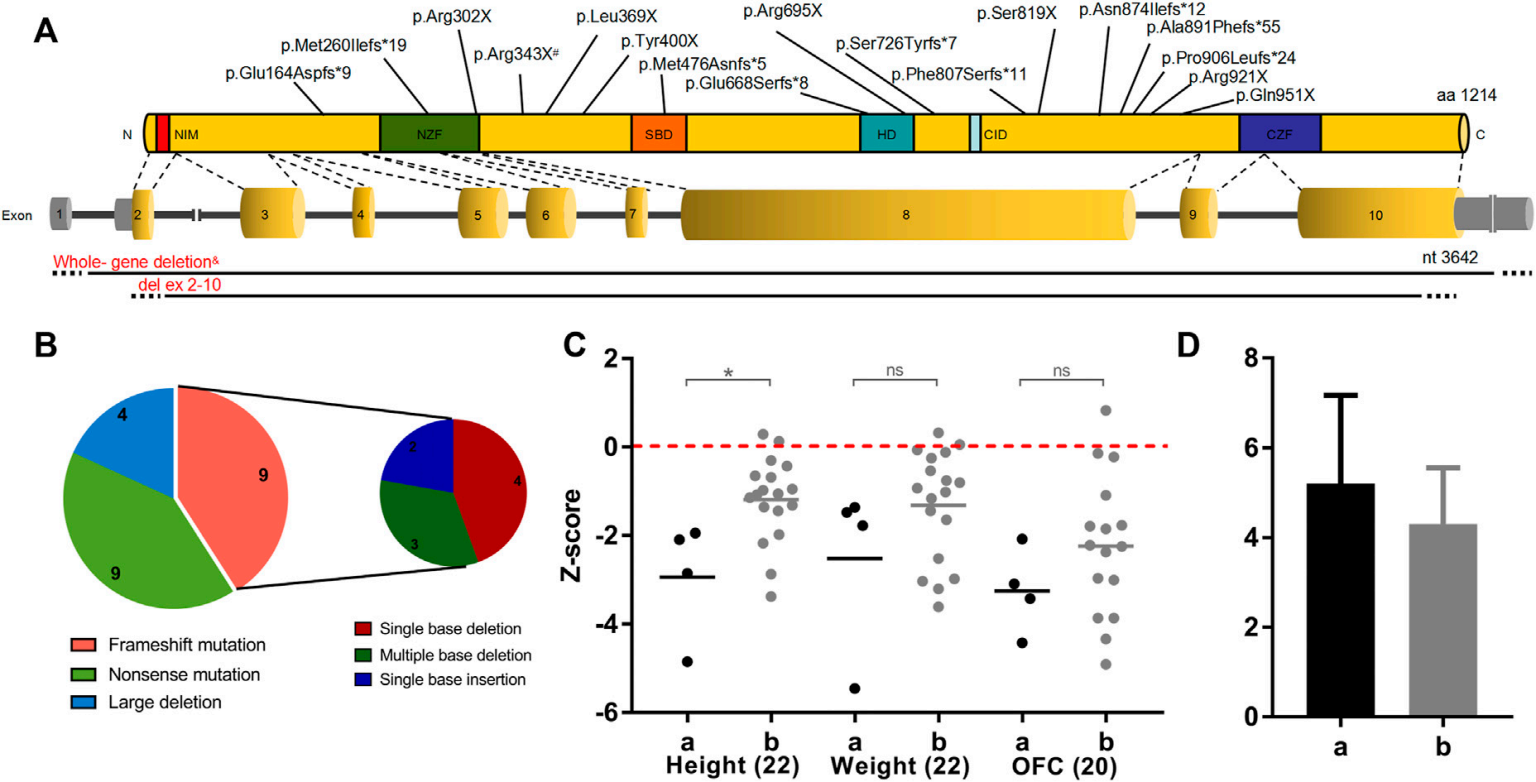
*ZEB2* variations and the genotype-phenotype correlation with clinical features. **(A)** Position of genetic defects identified in our cohort on a schematic representation of the *ZEB2* transcript and protein. Red represents Group a with large deletion (*n* = 4), black represents Group b with intragenic nonsense/frameshift variant (*n* = 18). ^#^Two patients had the same nonsense mutation (c.1027C>T) of *ZEB2*, and ^&^three patients had the whole *ZEB2* gene deletion (0.35–6.85 Mb). **(B)** Distribution of the different *ZEB2* defects found in our cohort. **(C)** Correlation between physical development (height, weight and OFC) and broad categories of the underlying heterozygous *ZEB2* variants. Student’s t test was performed to compare between groups. *p < 0.05, ns, not significant. OFC, occipital frontal circumference. **(D)** Number of involved systems in relation to broad categories of the underlying *ZEB2* variants.

Overall, twenty-two patients were reported, ranging in age from 1 to 10 years (Tables 1, 2). The mean age at the last clinical evaluation was 3 years and 6 months. The male-to-female ratio was 12:10. All patients were sporadic, and all variants were *de novo*, heterozygous and classified as pathogenic according to the ACMG criteria. No consanguinity was reported. Nine patients were discovered to carry frameshift variants (40.9%), including small deletions or insertions. Nine patients had nonsense variants (40.9%), with two of them carrying the same point mutation (c.1027C>T, p.Arg343X). In addition, chromosome deletions encompassing the *ZEB2* gene were found in four patients (18.2%), among which three cases were affected by whole gene deletion (0.35–6.85 Mb), and the latter was affected by intragenic deletion comprising exons 2–10 (0.13 Mb). The genetic and molecular study results are summarized in Figures 1A,B and Table 3.

**TABLE 1:** Main clinical features of the cohort.

Item	Our cohort	Total, N = 22 (%)
Gender	Male	12 (54.5)
	Female	10 (45.5)
Age (years)	1–3	13 (59.1)
	3–6	7 (31.8)
	6–10	2 (9.1)
Gestational week	<37	3 (13.6)
	37–42	19 (86.4)
Birth weight (g)	<2500	2 (9.1)
	2500–4000	18 (81.8)
	>4000	2 (9.1)
Mode of delivery	Spontaneous delivery	11 (50)
	Caesarean section	11 (50)
Height centile	<3rd	8 (36.4)
	3rd–10th	3 (13.6)
	10th-25th	6 (27.3)
	25–50th	3 (13.6)
	50–75th	2 (9.1)
Weight centile	<3rd	6 (27.3)
	3rd–10th	5 (22.7)
	10th–25th	5 (22.7)
	25–50th	4 (18.2)
	50–75th	2 (9.1)
OFC centile	<3rd	15 (68.2)
	3rd–10th	2 (9.1)
	10th–25th	1 (4.5)
	25–50th	2 (9.1)
	50–75th	1 (4.5)
	75–90th	1 (4.6)
Clinical feature	HSCR	4 (18.2)
	Epilepsy	16 (72.7)
	CHD	13 (59.1)
	Urogenital/renal	8 (36.4)

Note: OFC, occipital frontal circumference; HSCR, hirschsprung disease; CHD, congenital heart disease.

**TABLE 2:** Clinical characteristics of twenty-two patients with Mowat-Wilson syndrome.

Patient ID	Gender	Age at last examination	Gestational week	Birth weight (g)/centile	Present height (cm)/centile	Present weight (kg)/centile	Present OFC (cm)/centile	HSCR	Epilepsy	CHD	Urogenital/renal anomaly
1	F	2y4m	41	3100/25–50th	89/25–50th	9.2/<3rd	45/<3rd	−^b^	−	PFO	—
2	M	4y2m	39–40	3300/25–50th	94/<3rd	11.5/<3rd	45/<3rd	+	+	LSVC	—
3	M	2y10m	37–38	3800/75–90th	91/3rd–10th	13.5/25–50th	48/10–25th	+	+	PDA, VSD	Hypospadias
4	F	1y9m	41	4010/>90th	81/10–25th	11/25–50th	48/75–90th	+	−	ASD	—
5	M	3y1m	39–40	3300/25–50th	90/<3rd	10.5/<3rd	43.5/<3rd	−	+	PDA, PFO	Cryptorchidism, micropenis, pyelic separation
6	M	2y3m	37–38	3050/10–25th	90/25–50th	12/10–25th	45/<3rd	−	+	—	Hypospadias
7	F	1y7m	37–38	2540/<3rd	74/<3rd	9.0/3rd–10th	44/<3rd	−	+	PFO, TR	—
8	M	1y8m	39–40	3080/25–50th	74/<3rd	8.9/<3rd	43/<3rd	−^b^	−	PDA, ASD	Hypospadias, penoscrotal transposition
9	F	2y0m	37–38	2750/3rd–10th	85/25–50th	10/3rd–10th	45/<3rd	−	+	PDA, PFO	—
10	M	2y1m	37–38	3650/75–90th	73/<3rd	7.1/<3rd	43/<3rd	+	−	—	—
11	F	3y2m	39–40	3130/25–50th	92/3rd–10th	10.5/<3rd	45/<3rd	−	+	PDA, VSD, ASD, PAS	—
12	F	5y5m	39–40	3180/25–50th	104/<3rd	16/3rd–10th	46/<3rd	−	+	—	—
13	M	2y0m	39–40	4008/>90th	89/50–75th	13/50–75th	45.4/<3rd	−	+	—	—
14	F	2y9m	39–40	4000/>90th	90/10–25th	12.5/10–25th	48/25–50th	−	−	PFO	Pyelic separation
15	M	7y7m	34–36	3500/50–75th	122/10–25th	22/10–25th	47.2/<3rd^a^	−	+	—	—
16	M	1y8m	39–40	2660/3rd–10th	80/3rd–10th	10/3rd–10th	43/<3rd	−	+	—	Short prepuce
17	F	4y8m	34–36	2460/<3rd	100/<3rd	15/3rd–10th	46/<3rd	−	+	—	—
18	F	2y8m	39–40	3630/75–90th	90/10–25th	13.5/50–75th	48/25–50th	−	+	PFO, VSD	—
19	M	2y4m	37–38	2670/3rd–10th	84/<3rd	12/10–25th	46/<3rd	−	−	PDA, PFO	Webbed penis
20	F	5y5m	41	3200/25–50th	110/10–25th	19/25–50th	48/3rd–10th	−	+	VSD, ASD	—
21	M	5y6m	34–36	2850/10–25th	110/10–25th	17.5/10–25th	49/3rd–10th	+	+	—	Hypospadias
22	M	10y1m	39–40	3900/>90th	142/50–75th	33/25–50th	54/50–75th^a^	−	+	—	—

Note: OFC, occipital frontal circumference; HSCR, hirschsprung disease; CHD, congenital heart disease; y, years; m, months; d, days; PFO, patent foramen ovale; LSVC, left superior vena cava; PDA, patent ductus arteriosus; VSD, ventricular septal defect; ASD, atrial septal defect; TR, tricuspid regurgitation; PAS, pulmonary artery sling.

^a^ The US OFC growth reference was used.

^b^ Constipation.

**TABLE 3:** Molecular analysis of the *ZEB2* gene in our cohort.

Patient ID	Exon	Method	Type of variant	Gene mutation	Protein mutation
1	8	WES	Intragenic frameshift	c.2417delT	p.Phe807Serfs*11
2	8	WES	Intragenic nonsense	c.1200T>A	p.Tyr400X
3	8	Sanger sequencing	Intragenic nonsense	c.1027C>T	p.Arg343X
4	8	Sanger sequencing	Intragenic frameshift	c.2621delA	p.Asn874Ilefs*12
5	8	WES	Intragenic nonsense	c.2456C>G	p.Ser819X
6	8	WES	Intragenic frameshift	c.2002delG	p.Glu668Serfs*8
7	2–10	WES	Large deletion	chr2:145147017-145274917del	—
8	5	WES	Intragenic frameshift	c.492_517delGGAGTACCTTCAGCGC	p.Glu164Aspfs*9
				AGTGACACAG	
9	6	WES	Intragenic frameshift	c.779dupT	p.Met260Ilefs*19
10	1–10	WES	Large deletion	chr2:141213978-148010654del	—
11	8	WES	Intragenic nonsense	c.2083C>T	p.Arg695X
12	1–10	WES	Large deletion	chr2:145000000-145351228 del	—
13	7	WES	Intragenic nonsense	c.904C>T	p.Arg302X
14	8	WES	Intragenic frameshift	c.2712delT	p.Pro906Leufs*24
15	8	WES	Intragenic frameshift	c.2670_2677delTGCCAAAC	p.Ala891Phefs*55
16	8	WES	Intragenic frameshift	c.2177_2180delCTTT	p.Ser726Tyrfs*7
17	1–10	aCGH	Large deletion	chr2:138434153-145285163 del	—
18	8	WES	Intragenic nonsense	c.2851C>T	p.Gln951X
19	8	WES	Intragenic frameshift	c.1426dupA	p.Met476Asnfs*5
20	8	WES	Intragenic nonsense	c.2761C>T	p.Arg921X
21	8	WES	Intragenic nonsense	c.1027C>T	p.Arg343X
22	8	WES	Intragenic nonsense	c.1106_1115delinsAG	p.Leu369X

Note: WES, whole exome sequencing; aCGH, array-based comparative genomic hybridization. Red highlights patients in Group a with large deletions (*n* = 4).

Variants in the current study were stratified into two groups: a, large deletion, including all coding exons of a *ZEB2* allele (*n* = 4); b, intragenic nonsense/frameshift variant, including nonsense and frameshift mutations within *ZEB2* (*n* = 18). Based on these categories, we further delineated the clinical features in our patients.

### Genotype-phenotype correlations with physical development

As shown in Table 1, both the height and weight of all twenty-two patients at the last examination were lower than the 75th percentile, with only 9% higher than the 50th percentile (2 in 22 patients) (Li et al., 2009; Zong and Li, 2013). Because the Chinese OFC (occipital frontal circumference) standard is set for children younger than 6 years old, the approximate percentile of two older MWS patients aged 7 years (<3rd) and 10 years (50–75th) were estimated with the US OFC growth reference chart (Rollins et al., 2010) and not included in the following statistical analysis of the Z score value for consistency. Only two MWS OFCs were beyond the 50th percentile, and 72.7% of cases were microcephalic (16 of 22 patients, at least 2 SDs below the mean). After adjustment for age, Group b showed better status (Figure 1C). The mean Z score values of all 3 indices in Group a were lower than those in Group b, with statistical significance detected in height. In addition, the average number of involved systems in Group a was 5.2, which was higher than that in Group b (4.5) (Figure 1D).

### Genotype-phenotype correlations with neurodevelopment

**FIGURE 2:**
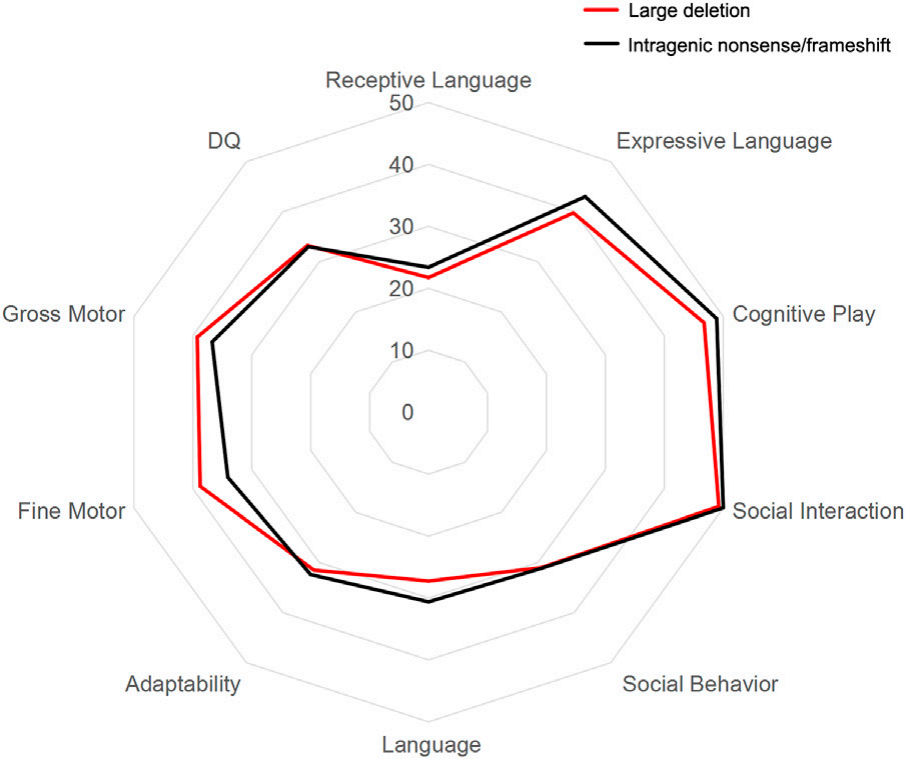
Results of Gesell Developmental Schedules (GDS) and Diagnostic Receptive and Expressive Assessment of Mandarin-Infant & Toddler (DREAM-IT) assessment in MWS patients. The mean scores of the general and subscale quotients of GDS and DREAM-IT assessment in two groups are shown. Red represents Group a with large deletion (*n* = 4), black represents Group b with intragenic nonsense/frameshift variant (*n* = 18).

The GDS was applied to evaluate the mental development of each child with MWS. The average quotients of twenty-two MWS patients are listed in Table 4. The developmental quotients of all five domains were less than 40 points, which were comprehensively delayed, especially language. According to the variant categories, the developmental quotients in Group a were lower than those of Group b, except for the fine motor and gross motor subscale (Figure 2).

**TABLE 4:** Developmental quotients of the GDS and DREAM-IT (mean ± SD).

GDS	DQ	DREAM-IT	DQ
Gross motor	37.18 ± 15.52	Receptive language	23.09 ± 12.52
Fine motor	34.91 ± 13.00	Expressive language	42.41 ± 13.42
Adaptability	32.23 ± 13.54	Cognitive play	48.50 ± 15.44
Language	30.00 ± 12.15	Social interaction	49.86 ± 20.50
Social behavior	31.09 ± 10.59	—	—

Note: GDS, gesell developmental schedules; DQ, developmental quotient; DREAM-IT, diagnostic receptive and expressive assessment of mandarin-infant & toddler.

### Genotype-phenotype correlations with language development

**FIGURE 3:**
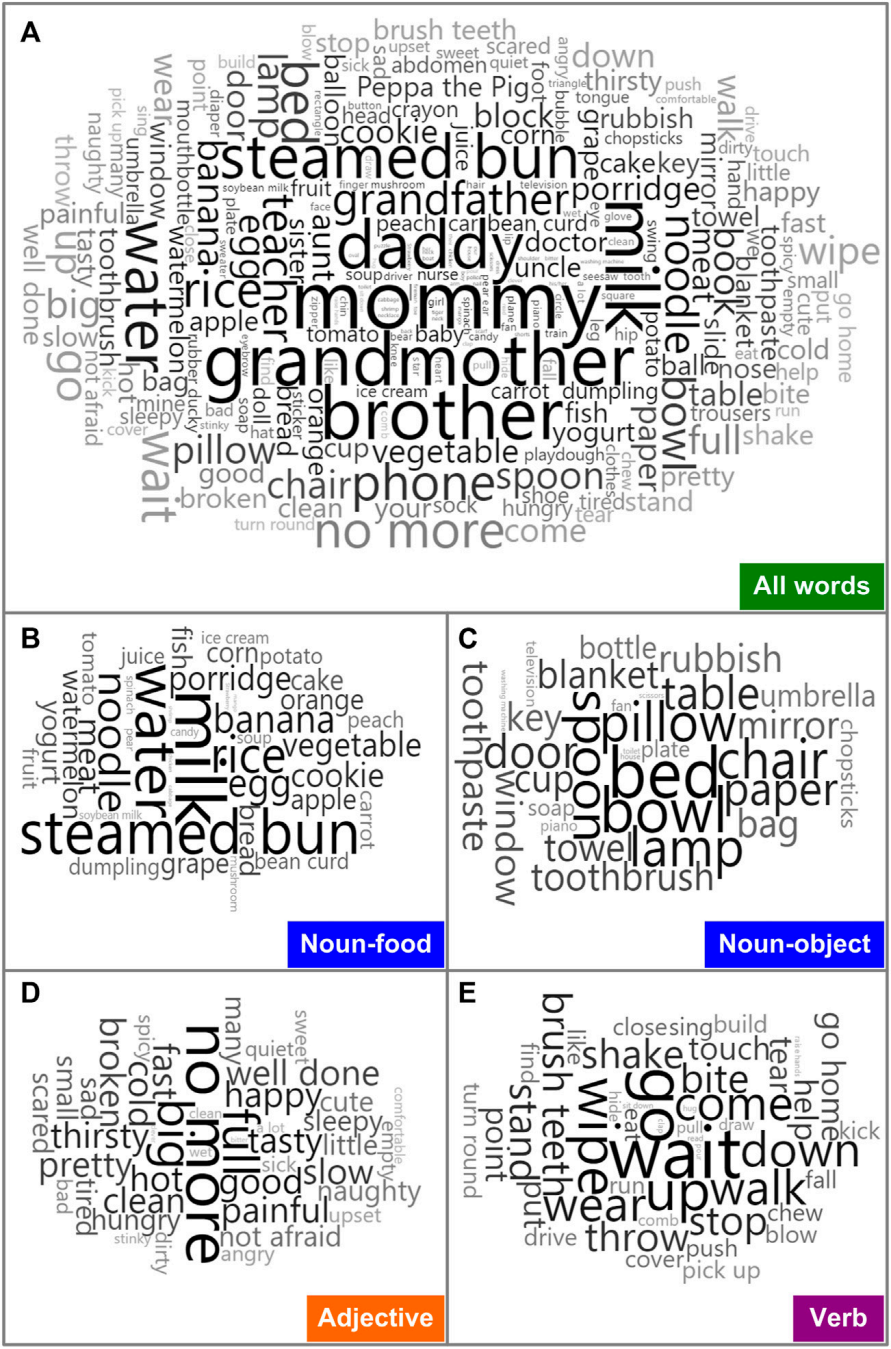
Representative words most understood by MWS patients in our study. Word cloud analysis of all **(A)** and summary categories for nouns **(B,C)**, adjectives **(D)** and verbs **(E)** using R package (wordcloud2, version 0.2.1) shows the most frequently occurring words. Font size denotes the frequency in each panel.

DREAM-IT tests were conducted to assess the language development of all MWS children. As shown in Table 4, the scores of the four dimensions were all less than 80, indicating developmental delay, especially in receptive language function. In the receptive language area, the types of words that MWS children understood most were nouns, followed by adjectives and verbs. The word cloud analysis showed the most frequently occurring words in each category, including person (mommy, grandmother, brother), food (milk, steamed bun, water), common objects (bed, bowl, lamp), verbs (go, wait, wipe) and adjectives (no more, big, full) (Figure 3). According to the variant categories, the developmental scores of all four dimensions in Group a were lower than those in Group b (Figure 2).

## Discussion

MWS is caused by deleterious *de novo* heterozygous variations in the *ZEB2* gene (Garavelli et al., 2017). The majority of variants lead to haploinsufficiency through premature stop codons or large deletions and thus can be conceived as *in vivo* models of the consequence of protein dysfunction on human neurodevelopment (Birkhoff et al., 2021). Moderate to severe intellectual disabilities are always present in MWS patients, accompanied by varying extents of language impairment (Garavelli et al., 2017; Ivanovski et al., 2018). A clear genotype-phenotype correlation has not yet been established. Our study attempted to comprehensively investigate physical, language and neurodevelopment in the largest MWS cohort of Chinese ancestry to date.

The average developmental quotients of twenty-two MWS patients evaluated by the GDS of all five domains were less than 40 points, suggesting a severe comprehensive delay, especially in language with the lowest quotient score. The *ZEB2* gene might be particularly important in language/vocal learning, in addition to other roles in neurodevelopment. We hope the present study will not remain just another endpoint of research and instead act as a starting point to further explore its role in language and the underlying molecular mechanism. The DREAM-IT test can provide four-dimensional analysis of language ability. Of note, all of our patients were demonstrated to have developmental delays, with a score lower than 80. In the receptive language area, the words that children with MWS understood consist of various types, including nouns, verbs, and adjectives. However, in expressive language, they can only speak a few words or emit only some vowels or consonants. According to previous studies (Ivanovski et al., 2018; Ho et al., 2020), receptive language skills were comparatively preserved in MWS patients, especially in those with speech absence. Surprisingly, the score of receptive language was the lowest among the others, at 23.09 ± 12.52. This is related to the rapid development of receptive language compared with the expressive development of normal children at an early age (Luinge et al., 2006). Although the receptive language of children with MWS was comparatively preserved, their actual ability fell behind the normal range when the developmental age of receptive language was compared with the chronological age, resulting in a relatively low developmental quotient score. In addition, there are several objective factors that can limit the accurate assessment of language development in MWS patients: 1) neurodevelopmental profiles are not necessarily reported from standardized clinical protocols for each affected child; 2) measurement of the expressive ability can be very difficult in those with minimal verbal skills, such as MWS patients; and 3) it is possible that the sociability of many individuals with MWS leads to the impression of relative receptive strength, but this may not be the case in actuality.

In comparison with expressive language, receptive language was a more effective manner of communication between children with MWS and their caregivers. In addition, receptive language training, such as simple instructions or gestures, was more easily performed for parents compared to the former. It should be noted that the scores of cognitive play and social communication in our cohort were higher than those of receptive and expressive language (48.50 ± 15.44 and 49.86 ± 20.50 versus 23.09 ± 12.52 and 42.41 ± 13.42, respectively). Playing and social communication can effectively encourage children to receive and study language. The relatively higher cognitive play and social communication abilities revealed here should thus be stressed to help caregivers conduct language studies of MWS patients. In humans, accelerated genomic regions (ARs, the fastest-evolving sequences in the genome) are postulated as having neurological functions, potentially related to the evolution of larger brains and language (Whalen and Pollard, 2022). ARs have been identified as enriched in noncoding regions near genes with known speech functions in humans and vocal learning birds, including *ZEB2*, and this convergent evolution might be an indication of the particular importance of *ZEB2* in vocal acquisition (Kamm et al., 2013; Oksenberg et al., 2013; Boyd et al., 2015; Cahill et al., 2021). However, the underlying mechanism of *ZEB2* involvement in language development is still unknown.

*ZEB2*, a zinc finger transcription factor, plays an essential role during embryonic development. It is expressed throughout the central nervous system, from the mesencephalon to the spinal cord, indicating an important participation in the process of neurogenic and gliogenic specialization. To date, no obvious genotype-phenotype association has been established in MWS patients, but cases with large deletions tend to be correlated with more severe phenotypes (Zweier et al., 2006). In the current study, whole or partial gene deletion of *ZEB2* was identified in 4 patients and assigned to Group a. Compared with the other 18 children in Group b, these patients exhibited more serious delays in physical, language and neurodevelopment, together with an increased severity with multiple systems affected, confirming the previous conclusion. The language ability of all patients within Group a exhibited a worse status in all four dimensions, and the three body indices were also lower than those of Group b, although at a limited sample size. Ivanovski’s study indicated that milder clinical presentations could be identified with variant *ZEB2* proteins that were predicted to preserve some functionality (Ivanovski et al., 2018). Some researchers suspected that the defective protein may influence the expression level of the wild-type allele in a dominant-negative fashion. Others have debated that the dominant-negative fashion may be partial and not devastating for various reasons, such as less stability of the defective protein or the potential partial compensation of the loss of protein from the mutant allele (Rossi et al., 2015). Anyhow, more studies are needed to better understand the genotype-phenotype association, particularly correlating formal assessments of ID, speech or other phenotypes with functional genomic studies of these atypical variants in *ZEB2*.

In conclusion, our study delineated the phenotypic spectrum of the largest MWS cohort in China and provided comprehensive profiling of their physical, language and neurodevelopment features, which will provide important guidance for appropriate intervention and patient care in the future. The scarcity of functional data makes it difficult to establish genotype–phenotype correlations and assess their statistical significance, and further elucidation of this would require a deeper molecular analysis of the mutant *ZEB2* function.

## Data Availability

The original contributions presented in the study are included in the article/Supplementary Materials, further inquiries can be directed to the corresponding authors.
